# HIV-PULSE: a long-read sequencing assay for high-throughput near full-length HIV-1 proviral genome characterization

**DOI:** 10.1093/nar/gkad790

**Published:** 2023-10-11

**Authors:** Laurens Lambrechts, Noah Bonine, Rita Verstraeten, Marion Pardons, Ytse Noppe, Sofie Rutsaert, Filip Van Nieuwerburgh, Wim Van Criekinge, Basiel Cole, Linos Vandekerckhove

**Affiliations:** HIV Cure Research Center, Department of Internal Medicine and Pediatrics, Ghent University Hospital, Ghent University, 9000 Ghent, Belgium; BioBix, Department of Data Analysis and Mathematical Modelling, Faculty of Bioscience Engineering, Ghent University, 9000 Ghent, Belgium; HIV Cure Research Center, Department of Internal Medicine and Pediatrics, Ghent University Hospital, Ghent University, 9000 Ghent, Belgium; BioBix, Department of Data Analysis and Mathematical Modelling, Faculty of Bioscience Engineering, Ghent University, 9000 Ghent, Belgium; HIV Cure Research Center, Department of Internal Medicine and Pediatrics, Ghent University Hospital, Ghent University, 9000 Ghent, Belgium; BioBix, Department of Data Analysis and Mathematical Modelling, Faculty of Bioscience Engineering, Ghent University, 9000 Ghent, Belgium; HIV Cure Research Center, Department of Internal Medicine and Pediatrics, Ghent University Hospital, Ghent University, 9000 Ghent, Belgium; HIV Cure Research Center, Department of Internal Medicine and Pediatrics, Ghent University Hospital, Ghent University, 9000 Ghent, Belgium; HIV Cure Research Center, Department of Internal Medicine and Pediatrics, Ghent University Hospital, Ghent University, 9000 Ghent, Belgium; Laboratory of Pharmaceutical Biotechnology, Faculty of Pharmaceutical Sciences, Ghent University, 9000 Ghent, Belgium; BioBix, Department of Data Analysis and Mathematical Modelling, Faculty of Bioscience Engineering, Ghent University, 9000 Ghent, Belgium; HIV Cure Research Center, Department of Internal Medicine and Pediatrics, Ghent University Hospital, Ghent University, 9000 Ghent, Belgium; HIV Cure Research Center, Department of Internal Medicine and Pediatrics, Ghent University Hospital, Ghent University, 9000 Ghent, Belgium

## Abstract

A deep understanding of the composition of the HIV-1 reservoir is necessary for the development of targeted therapies and the evaluation of curative efforts. However, current near full-length (NFL) HIV-1 proviral genome sequencing assays are based on labor-intensive and costly principles of repeated PCRs at limiting dilution, restricting their scalability. To address this, we developed a high-throughput, long-read sequencing assay called HIV-PULSE (HIV Proviral UMI-mediated Long-read Sequencing). This assay uses unique molecular identifiers (UMIs) to tag individual HIV-1 genomes, allowing for the omission of the limiting dilution step and enabling long-range PCR amplification of many NFL genomes in a single PCR reaction, while simultaneously overcoming poor single-read accuracy. We optimized the assay using HIV-infected cell lines and then applied it to blood samples from 18 individuals living with HIV on antiretroviral therapy, yielding a total of 1308 distinct HIV-1 genomes. Benchmarking against the widely applied Full-Length Individual Proviral Sequencing assay revealed similar sensitivity (11 vs 18%) and overall good concordance, although at a significantly higher throughput. In conclusion, HIV-PULSE is a cost-efficient and scalable assay that allows for the characterization of the HIV-1 proviral landscape, making it an attractive method to study the HIV-1 reservoir composition and dynamics.

## Graphical Abstract

**Graphical Abstract:**
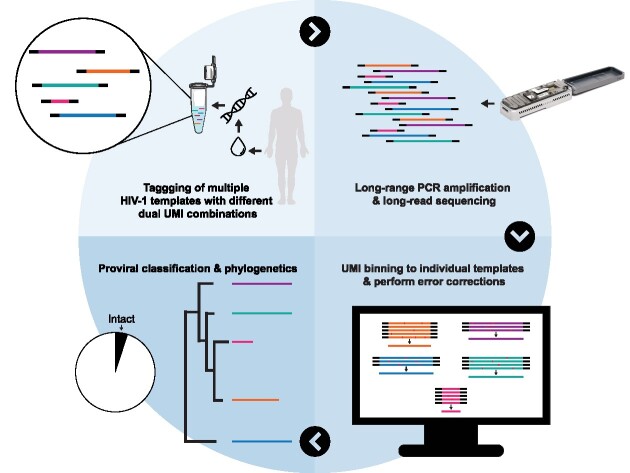

## Introduction

The establishment of a viral reservoir shortly after HIV-1 infection leads to long-term viral persistence in people living with HIV-1 (PLWH) (1–3). While antiretroviral therapy (ART) can successfully suppress viral replication, it is not curative as the viral reservoir is not targeted (4,5). Consequently, lifelong adherence to ART is required to prevent viral rebound, which usually takes place within several weeks following ART cessation (6). Despite the relatively low frequency of infected CD4 T cells that remain during ART (1/1000–1/10 000), the size of the viral reservoir is remarkably stable, with an estimated half-life of 44 months (7,8). The search for curative interventions targeting the viral reservoir remains one of the top priorities for achieving HIV-1 remission (9), however, this search is faced with two major challenges: (i) a lack of knowledge of the mechanisms governing HIV-1 latency and reservoir maintenance; and (ii) a lack of high-throughput methods to measure the efficacy of reservoir-reducing interventions. To address these problems, technological advances that allow for a deep and high-throughput reservoir characterization are urgently needed (10).

Historically, the qualitative assessment of the HIV-1 reservoir has been carried out using two main types of assay: (i) viral outgrowth assays (VOA), in which replication-competent viruses are reactivated and propagated*ex vivo* at limiting dilution, followed by quantification and sequencing of the cultured viral genomes (11,12); and (ii) sequencing-based assays, where single proviral genomes are PCR-amplified from bulk DNA at limiting dilution, followed by Sanger- or short-read next-generation sequencing (NGS) (13–19). The VOA-based methods have the inherent benefit that they focus on replication-competent viruses, although they are usually long, costly and labor-intensive and have been shown to underestimate the true size of the replication-competent fraction following one round of reactivation (15). Sequencing-based methods allow the assessment of a small subgenomic region of interest or the near full-length (NFL) proviral genome (∼90%) (13–19). In the case of the latter, the percentage of genome-intact proviruses can be derived, which has previously been estimated at 2–5% on average (16). More recently, several flow-cytometry-based assays have been developed to isolate and study HIV-infected cells harboring an inducible provirus such as Simultaneous TCR, Integration site and Provirus sequencing (STIP-Seq), which specifically targets the translation-competent reservoir (20–22). In these assays, the infected cells are dispensed into single wells of a microtiter plate, followed by genomic or transcriptomic sequencing.

A common denominator of the assays described here is that they all rely on the physical isolation of individual viral genomes into different wells of a microtiter plate, followed by the PCR amplification of each genome in separate reactions (23). This principle is both labor-intensive and costly, severely limiting the applicability in large scale projects.

Up until the advent of long-read sequencing technologies, long amplicons (>5 kb) were either sequenced by a series of overlapping Sanger sequencing reactions, or by fragmentation of the amplicon into smaller pieces followed by short-read NGS (16–18). Long-read sequencing technologies offer the ability to sequence long amplicons in a single read, however, these technologies suffer from a high error rate of single-pass reads (∼5–10%) (24). Recently, Karst *et al.* developed a protocol that uses unique molecular identifiers (UMIs) to obtain high-accuracy consensus sequences from long amplicons (>5 kb), overcoming the problem of the limited single-read accuracy (25).

Here, we present a new assay that allows for high-throughput amplicon sequencing of NFL HIV-1 genomes, called HIV-PULSE: HIV Proviral UMI-mediated Long-read Sequencing. By tagging individual HIV-1 genomes with two distinct UMIs, the step of limiting dilution can be omitted, enabling the amplification of many NFL genomes in a single reaction (25). After optimization of the assay on HIV-infected cell lines, we used the protocol to characterize the viral reservoirs of a cohort of PLWH treated during the chronic phase of infection (*n* = 18). Benchmarking against the widely used Full-Length Individual Proviral Sequencing (FLIPS) assay revealed comparable accuracy and efficiency, but a remarkably higher throughput and lower cost per sequenced NFL HIV-1 genome (17). In conclusion, HIV-PULSE is a valuable addition to the arsenal of HIV-1 proviral sequencing methods and opens new possibilities for investigating the composition and dynamics of the HIV-1 reservoir.

## Material and methods

### Study participants and sample collection

A total of 18 individuals on suppressive ART were included in this study (Supplementary Table S1). Participants were recruited at Ghent University Hospital and donated blood samples. Note that all included participants identify as male, resulting in a homogenous sex distribution not representative of the worldwide PLWH population. Some participants agreed to additional leukapheresis to harvest large amounts of leukocytes. Peripheral blood mononuclear cells were isolated by Ficoll density gradient centrifugation and were cryopreserved in liquid nitrogen. CD4 T cells were isolated from peripheral blood mononuclear cells by negative selection using the EasySep Human CD4 T Cell Enrichment Kit (StemCell Technology, catalog no. 19052). All participants signed informed consent forms approved by the Ethics Committee of the Ghent University Hospital (Belgium) (Ethics Committee Registration numbers 2015/0894, 2016/0457, BC-07056).

### Cell lines

J-Lat 8.4 cells (ARP-9847, contributed by E. Verdin), a Jurkat-based cell line latently infected with HIV, and Jurkat E6.1 cells (ARP-177, contributed by A. Weiss) were obtained through the NIH HIV Reagent Program, Division of AIDS, NIAID and NIH (26,27). J-Lat and Jurkat cells were grown in RPMI1640 (Gibco, catalog no. 11875093) supplemented with 10% fetal bovine serum (HyClone, catalog no. RB35947) and 1% Pen/Strep (Gibco, catalog no. 15140122).

### DNA isolation and HIV-1 DNA measurements

Genomic DNA from pelleted negatively selected CD4 T cells was isolated via column extraction using the DNeasy Blood & Tissue Kit (Qiagen, catalog no. 69506) according to the manufacturer's instructions. The DNA concentration of each extract was measured on a Qubit 3.0 fluorometer using the Qubit dsDNA BR assay kit (ThermoFisher Scientific, catalog no. Q32853). The HIV-1 copy number was determined by a total HIV-1 DNA assay on droplet digital PCR (Bio-Rad, QX200 system), as described previously (28). PCR amplification was carried out with the following cycling program: 10 m at 95°C; 40 cycles (30 s at 95°C, 1 m at 56°C); 10 m at 98°C. Droplets were read on a QX200 droplet reader (Bio-Rad). Analysis was performed using ddpcRquant software (29). A multiplex dPCR assay (QIAcuity, Qiagen), measuring four different genomic regions (primers listed in Supplementary Table S2), was used to assess PCR amplification of HIV-1 template using the following cycling program: 2 min at 95°C; 40 cycles (30 s at 94°C, 1 min at 56°C) (30,31). Analysis was performed using the QIAcuity Software Suite (v.2.1.7.182, Qiagen).

### Full-length individual proviral sequencing

The Full-Length Individual Proviral Sequencing (FLIPS) assay was performed as described by Hiener *et al.* (17). A detailed protocol can be found on the following link: dx.doi.org/10.3791/58016. In short, genomic DNA from negatively selected CD4 T cells was used as input for a nested PCR performed at an endpoint dilution where <30% of the reactions are positive. The cycling conditions were 94°C for 2 m; then 94°C for 30 s, 64°C for 30 s, 68°C for 10 m for 3 cycles; 94°C for 30 s, 61°C for 30 s, 68°C for 10 m for 3 cycles; 94°C for 30 s, 58°C for 30 s, 68°C for 10 m for 3 cycles; 94°C for 30 s, 55°C for 30 s, 68°C for 10 m for 21 cycles; then 68°C for 10 m. For the second round, 10 extra cycles at 55°C were included. The PCR products were visualized using agarose gel electrophoresis (1% agarose gel). Proviral amplicons from positive wells were cleaned using AMPure XP beads (Beckman Coulter, catalog no. A63880), followed by a quantification of each cleaned provirus with Quant-iT PicoGreen dsDNA Assay Kit (Invitrogen, catalog no. P11496).

### Illumina short-read sequencing

Selected FLIPS proviral amplicons underwent NGS library preparation using the Nextera XT DNA Library Preparation Kit (Illumina, catalog no. FC-131–1096) with indexing of 96-samples per run according to the manufacturer's instructions (Illumina, catalog no. FC-131–2001), except that input and reagents volumes were halved and libraries were normalized manually. The pooled library was sequenced on a MiSeq Illumina platform via 2 × 150 nt paired-end sequencing using the 300 cycle v.2 kit (Illumina, catalog no. MS-102–2002).

### HIV-PULSE assay methodology

#### Pre-amplification

**Figure 1.**
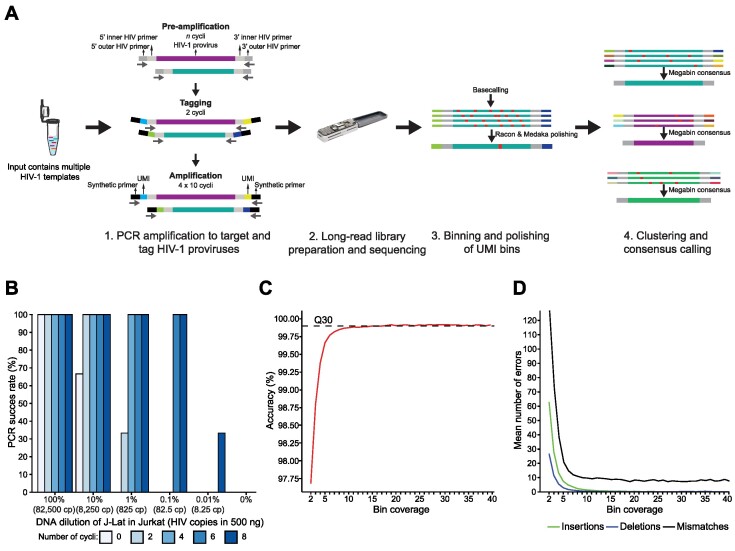
HIV-PULSE methodology overview and performance evaluation. **(A)** Schematic overview of the HIV-PULSE assay. A PCR reaction with bulk DNA containing multiple HIV-1 templates is pre-amplified using outer HIV-1 primers for a limited number of cycles to improve sensitivity. Next, pre-amplified material is tagged with a dual barcode consisting of a unique molecular identifier (UMI) attached to both ends using an HIV-1 specific inner primer. To generate enough material for long-read library preparation, the tagged material is amplified with synthetic primers in several PCR rounds followed by clean up to prevent length bias. The illustration of the microtube was obtained from SMART (Servier Medical Art; http://smart.servier.com/), licensed under a Creative Common Attribution 3.0 Unported license (https://creativecommons.org/licenses/by/3.0/). **(B)** Success rate of the HIV-PULSE assay for different input ratios of HIV-1 with varying number of PCR cycles during pre-amplification. Each data point represents the mean of triplicate experiments for each condition. **(C)** Mean accuracy of HIV-PULSE bin sequences with increasing bin coverage compared to the Illumina reference sequence. The dashed line indicates the Q30 (99.9% accuracy) threshold. **(D)** Mean number of errors (insertions, deletions and mismatches) found in HIV-PULSE bin sequences of 9.5 kb with increasing bin coverage compared to the Illumina reference sequence.

A first PCR was used to specifically target and pre-amplify HIV-1 proviral templates using the outer primers of a nested HIV-1 primer set (designed by Pinzone *et al.*, listed in Supplementary Table S2, Figure 1A) (19). Each PCR reaction contained 500 ng of genomic DNA (maximum amount of input template recommended by the manual), 2 μl of LongAmp Taq DNA Polymerase (NEB, catalog no. M0323L), 0.5 μM of each primer (First PCR F, First PCR R), 1.5 μl of 10 mM dNTPs (Promega, catalog no. C1141), 10 μl of 5× LongAmp Taq Reaction Buffer in 50 μl. The following cycling conditions were used: 94°C for 1 m 15 s; then 94°C for 30 s, 63°C for 30 s, 65°C for 10 m for six cycles; then 65°C for 10 m. The number of amplification cycles can be reduced to five cycles for samples from individuals with a high reservoir size (>2500 total HIV-1 DNA copies/million CD4) to prevent overbinning. PCR products were cleaned using CleanPCR magnetic beads (CleanNA, catalog no. CPCR-0050) at a 1.0× beads:sample ratio.

#### Tagging HIV-1 templates

A second PCR was performed to tag both ends of the pre-amplified proviral HIV-1 templates with a tailed UMI (listed in Supplementary Table S2). Primers were designed to contain: (i) a synthetic primer binding site used in later stages for amplification, (ii) a UMI with a repetitive pattern of 12 random nucleotides and 6 degenerate nucleotides (Y/R) and (iii) an HIV-1 inner primer of the nested primer set to target the pre-amplified templates (Figure 1A, Supplementary Figure S1A). Each PCR reaction contained all the cleaned pre-amplified product (30 μl), 2 μl of LongAmp Taq DNA Polymerase (NEB, catalog no. M0323L), 0.5 μM of each primer (Second PCR F UMI, Second PCR R UMI), 1.5 μl of 10 mM dNTPs (Promega, catalog no. C1141), 10 μl of 5× LongAmp Taq Reaction Buffer in 50 μl. The following cycling conditions were used: 94°C for 1 m 15 s; then 94°C for 30 s, 58°C for 30 s, 65°C for 10 m for two cycles; then 65°C for 10 m. Tagged PCR products were cleaned using CleanPCR magnetic beads (CleanNA, catalog no. CPCR-0050) in a custom buffer solution (based on the ‘SPRI size selection protocol for >1.5–2 kb DNA fragments’ protocol provided by Oxford Nanopore Technologies (ONT)) at a 0.9× beads:sample ratio and eluted in 30 μl of nuclease-free water.

#### Amplification of UMI-tagged proviruses

The next steps used four consecutive PCR amplification rounds each of 10 cycles followed by a clean up to produce enough template input required for long-read sequencing while preserving amplicon size distributions. Here, we made use of a primer set that binds to the synthetic binding site incorporated during the previous tagging stage. The PCR mix consists of 2 μl of LongAmp Taq DNA Polymerase (NEB, catalog no. M0323L), 0.5 μM of each primer (ncec_pcr_fw_v7, ncec_pcr_rv_v7), 1.5 μl of 10 mM dNTPs (Promega, catalog no. C1141), 10 μl of 5× LongAmp Taq Reaction Buffer in 50 μl. For the first PCR amplification round all the cleaned tagging products from the previous step (30 μl) were used as template inputs while during the second, third and fourth amplification rounds only one-third of the cleaned product of the previous round is used (10 μl). The following cycling conditions were used: 94°C for 1 m 15 s; then 94°C for 30 s, 58°C for 30 s, 65°C for 10 m for 10 cycles; then 65°C for 10 m. PCR products were cleaned after each consecutive round using regular CleanPCR magnetic beads (CleanNA, catalog no. CPCR-0050) at a 1.0× beads:sample ratio and eluted in 30 μl of nuclease-free water. During the last round of 10 cycles, the regular primers were switched for a custom set of tailed primers to barcode the PCR products from the same participant with a specific, identical identifier (listed in Supplementary Table S2). After the last PCR round, the end products were visualized using agarose gel electrophoresis (1% agarose gel) and the DNA concentration was determined using a Qubit 3.0 fluorometer with the Qubit dsDNA BR assay kit (ThermoFisher Scientific, catalog no. Q32853).

### ONT long-read sequencing

Samples were multiplexed using the Native Barcoding Kit (ONT, catalog no. EXP-NBD104) using the following strategy: each PCR replicate was assigned to a different ONT barcode and contained equimolarly pooled PCR products from different participants. In later stages, this allows the assignment of reads to the correct PCR replicate by the ligated ONT barcode and to the correct sample by the participant-specific identifier attached during the last PCR round (Supplementary Figure S1A). For library preparation, the Ligation Sequencing Kit (ONT, catalog no. SQK-LSK109) was used following the manufacturer's instructions. Samples were sequenced on a MinION ONT device using MinION R10.3 flow cells and the MinKNOW v.21.02.1 software followed by basecalling at super accuracy mode and demultiplexing with Guppy v.5.0.17. Estimated calculations regarding minimal HIV-PULSE sequencing depth requirements can be found in Supplementary Table S3.

### Bioinformatics analysis of long-read data

For the analysis of long-read data, a customized version of the UMI data analysis workflow as described by Karst *et al.* was used (Supplementary Figure S1B) (25). The adapted scripts can be found at https://github.com/laulambr/longread_umi_hiv, main changes include updated software versions of samtools (v.1.11), medaka (v.1.4.3) and racon (v.1.4.20). Before the data was analyzed using the UMI pipeline, the demultiplexed ONT reads were first mapped against the HXB2 reference sequence using minimap2 (2.17) to filter out non-HIV-1 reads. Next, the ‘longread_umi nanopore_pipeline’ workflow was run separately on each replicate read dataset using the following settings: -s 200 -e 200 -m 1500 -M 10000 -f CAAGCAGAAGACGGCATACGAGAT -F AAGTAGTGTGTGCCCGTCTGTTGTGTGAC -r AATGATACGGCGACCACCGAGATC -R GGAAAGTCCCCAGCGGAAAGTCCCTTGTAG -c 3 -p 1 -q r103_hac_g507 -U ‘r103_min_high_g360’. The workflow consists of the following consecutive steps: (i) trimming and filtering of the HIV-1 long-read sequencing data using Porechop (v.0.2.4, https://github.com/rrwick/Porechop), Filtlong (v.0.2.0, https://github.com/rrwick/Filtlong) and cutadapt (v.2.7) (32); (ii) extraction of UMI reference sequences using cutadapt (v.2.7) and usearch (32,33); (iii) binning of reads to UMI combinations using bwa (v.0.7.17) and samtools (v.1.11) while excluding chimeric artifacts; (iv) generation of bin centroid sequences using usearch and minimap2 (v.2.17) (33,34) and (v) polishing of bin centroid data by multiple rounds of racon (v.1.4.20) and a final round of Medaka (v.1.4.3, https://github.com/nanoporetech/medaka) (35).

Next, a custom bioinformatics workflow specific to the HIV-PULSE protocol was run to correct for pre-amplification, improve final bin accuracy and evaluate clonality among PCR replicates. First, the polished bin sequences from each replicate dataset were demultiplexed to their respective participant using the ‘longread_umi demultiplex’ workflow. For each participant, bin centroid sequences from all PCR replicates were pooled together and screened for the occurrence of identical UMIs. Identical UMI pairs in different PCR replicates are technically not possible but these artifacts may arise due to misassignment during the initial raw ONT reads demultiplexing by Guppy. In these cases, the bin in the replicate with the highest coverage was considered correct while the others were removed from their respective replicate datasets. As the assay relies on a pre-amplification phase, single proviral templates will have been amplified and potentially tagged into bins with different UMI pairs. This prohibits the screening for clonal proviruses present in one bulk PCR reaction as pre-amplification would also lead to the presence of identical proviruses in bins with different UMI pairs. To correct for this, identical proviruses (due to clonality or pre-amplification) present in the bin centroid sequences from each participant were identified by clustering genomes with similar sizes and >99.5% sequence identity into a megabin using usearch (33). For megabins consisting of more than three bins, a consensus sequence was constructed to help to resolve remaining errors. For megabins that only consisted of two bin centroid sequences, the bin with the highest coverage (∼accuracy) was retained while bins that did not cluster remained as unique bins. Finally, an assessment of clonality among different PCR replicates was made by screening the resulting megabins for the presence of bin sequences originating from different PCR replicates. If the same proviral sequence was found in multiple PCR replicates, it is considered as evidence of a potential clonal origin, as opposed to proviruses that are only found in one replicate. By performing multiple PCR replicates, one can identify clonal populations, however, accurate quantification of reservoir clonality is hindered by the fact that identical proviruses found within the same PCR replicate are collapsed and counted as one (to exclude potential bias by pre-amplification).

### Bioinformatics analysis of Illumina data

NFL genome sequences derived from FLIPS reactions were de novo assembled as follows: (i) FASTQ quality checks were performed with FastQC v.0.11.7 (http://www.bioinformatics.babraham.ac.uk/projects/fastqc) and removal of Illumina adaptor sequences and quality-trimming of 5′ and 3′ terminal ends was performed with bbmap v.37.99 (https://sourceforge.net/projects/bbmap/). (ii) Trimmed reads were de novo assembled using MEGAHIT v.1.2.9 with standard settings (36). (iii) Resulting contigs were aligned against the HXB2 HIV-1 reference genome using blastn v.2.7.1 with standard settings, and contigs that matched HXB2 were retained (37). (iv) Trimmed reads were mapped against the *de novo* assembled HIV-1 contigs to generate final consensus sequences based on per-base majority consensus calling, using bbmap v.37.99 (https://sourceforge.net/projects/bbmap/). Scripts concerning *de novo* assembly of HIV-1 genomes can be found at the following GitHub page: https://github.com/laulambr/virus_assembly.

### HIV-1 genome classification

NFL proviral genome classification was performed using the publicly available ‘Gene Cutter’ and ‘Hypermut’ webtools from the Los Alamos National Laboratory HIV sequence database (https://www.hiv.lanl.gov). Proviral genomes were classified in the following sequential order: (i) ‘Inversion’, the presence of internal sequence inversion, defined as region of reverse complementarity. (ii) ‘Large internal deletion’, internal sequence deletion of >1000 bp. (iii) ‘Hypermutated’, APOBEC-3G/3F-induced hypermutation. For each participant, a consensus sequence was generated from the remaining genomes and a new alignment with the consensus sequence as the reference was created. This alignment was then analyzed using the ‘Hypermut’ webtool, with proviruses scoring *P* < 0.05 considered hypermutated. (iv) ‘Packaging signal and/or major splice donor (PSI/MSD) defect’, proviruses containing a deletion >7 bp found in any of the four stem loops of the PSI region (SL1 (HXB2: 691–734), SL2 (HXB2: 736–754), SL3 (HXB2: 766–779) and SL4 (HXB2: 790–810)). This also includes the absence and/or point-mutation of both the MSD site (sequence GT, HXB2: 744–745) and the cryptic donor site (sequence GT, HXB2: 748–749) (19). Proviruses with a deletion covering PSI/MSD that extended into the *gag* gene, thereby removing the *gag* AUG start codon, were also classified into this category. (v) ‘Premature stop-codon/frameshift’, premature stop-codon or frameshift caused by mutation and/or sequence insertion/deletion in the essential genes *gag*, *pol* or *env*. Proviruses with insertion/deletion >49 nt in *gag*, insertion/deletion >49 nt in *pol*, or insertion/deletion >99 nt in *env* were also classified into this category. (vi) ‘Intact’, proviruses that displayed none of the above defects were classified into this category.

### Reference sequences

Proviral HIV-1 genomes from participants previously acquired with the FLIPS and STIP-Seq assays on negatively selected CD4 T cells in the context of former studies were included as references (20). Accuracy metrics on the new HIV-PULSE assay compared to FLIPS and STIP-Seq reference proviruses (sequenced using Illumina technology) were calculated using pomoxis (v.0.3.6, https://github.com/nanoporetech/pomoxis).

### Phylogenetic analysis

Sequences obtained with the HIV-PULSE, STIP-Seq and FLIPS assays were multiple aligned using MAFFT (v.7.471) (38). DIVEIN was used to calculate pairwise diversity among proviral sequences (39). Phylogenetic trees were constructed using PhyML v.3.0 (best of NNI and SPR rearrangements) and 1000 bootstraps (40). R (v.4.1.2), ggplot (v.3.3.5) and ggtree (v.3.2.1) were used for visualization and annotation of trees (41–43).

### Recombination analysis

To assess the occurrence of potential chimera formation, a result of template recombination during PCR, and to verify that our bioinformatics pipeline effectively filters out such artifacts, we performed HIV-PULSE on mixed genomic DNA from two different participants (P03 and P12_T1, 250 ng each). This experiment was performed in duplicate. PCR products were sequenced using the Ligation Sequencing Kit (ONT, catalog no. SQK-LSK109) on a MinION R9.4.1 flow cell. First, HIV-PULSE bins that passed the quality control of the bioinformatics pipeline were assigned to their respective participant using blastn (v.2.7.1) with a custom reference database that included a FLIPS-based consensus sequence for each participant (37). This assignment ensured that each proviral bin was attributed to its closest related participant. To further analyze potential recombination events, all the proviral HIV-PULSE bins were aligned to FLIPS sequences from the corresponding individuals, and a phylogenetic tree was constructed using PhyML v.3.0 (40). This tree allowed us to visually examine the relationships between the HIV-PULSE and the FLIPS reference sequences. In addition to the phylogenetic analysis, we employed the Recombination Detection Program (RDP4) with default settings to screen for evidence of recombination between FLIPS sequences and the mixed HIV-PULSE sequences (44). A possible recombination event was considered for manual inspection if it was predicted by at least five out of the seven available methods in RDP4.

### Statistics

Statistical analysis was performed with the R (v.4.1.2) software package and DIVEIN (39,41). Fisher's exact test was used to compare the proportions of sampled unique proviruses in the HIV-PULSE and FLIPS datasets for each participant. A Mann–Whitney *U*-test was used to compare size distributions of the observed proviral sequence lengths in the HIV-PULSE and FLIPS datasets. A Mann–Whitney *U-*test was used to compare the HIV-PULSE and FLIPS assay efficiencies. A Spearman correlation was used to investigate the relationship between the mean HIV-PULSE yield per PCR replicate and total HIV-1 DNA copies/million CD4 cells as measured by ddPCR. The normality of the data was determined using Shapiro–Wilk tests. For participant P12, an independent-samples *t*-test was used to compare the mean HIV-PULSE yield of distinct viruses per replicate between the two different timepoints. A two-sample *t*-test was performed using DIVEIN to compare the observed mean pairwise distance among intact sequences between the two timepoints.

## Results

### Optimization of HIV-PULSE experimental conditions

Since the frequency of HIV-1 infected cells in ART-suppressed PLWH is remarkably low, typically in the range of 100–1000 proviral genomes per million CD4 T cells, we set out to devise an assay allowing for the detection of such rare events using a UMI-binning strategy (Figure 1A) (7). By including a pre-amplification step, the sensitivity of the assay should considerably improve as more target templates are created, thus increasing the number of tagged templates transferred to the PCR amplification step. This hypothesis was tested by preparing a dilution series of J-Lat 8.4 DNA (HIV-infected CD4 T cell line) in Jurkat DNA (non-infected parental CD4 T cell line). The dilution series, ranging from 100 to 0.01% J-Lat 8.4 DNA (Jurkat as negative control), was subjected to either zero, two, four, six or eight cycles of pre-amplification, using primers that target NFL HIV-1 (Figure 1B). Each amplification was performed in triplicate, using a fixed input of 500 ng of genomic DNA (corresponding with ∼82 500 CD4 T cells) with the PCR success being determined by visualization of the ∼9.5 kb amplicon by agarose gel electrophoresis. As expected, reactions with an input of undiluted J-Lat DNA were all successful regardless of the number of pre-amplification cycles (Figure 1B). However, in samples with a proviral burden closer to those observed in samples from PLWH (∼0.1%) at least six cycles of pre-amplification were required for guaranteed PCR success.

To evaluate whether the addition of an overhanging end to the tagging primers (which includes sequences containing the UMI and amplification regions) influences the sensitivity of the assay, we determined the efficiency of the tagging primers compared to primers that did not have the overhanging tails. For this experiment, 250 ng of a 10% dilution of J-Lat 8.4 DNA in Jurkat DNA was used as input for the second tagging PCR performed with either the tailed primers or the non-tailed primers. Each condition was repeated in three replicates and HIV-1 copy numbers of the amplified products were measured by a multiplex HIV-1 dPCR. Comparing the fold changes in HIV-1 copy number increase after tagging revealed no appreciable differences in the efficiency of the primers with additional sequences compared to the primers without the additional sequences (Supplementary Figure S2), indicating that the overhangs have a negligible effect on tagging efficiency.

We then determined the most optimal PCR cycling conditions to amplify tagged proviral sequences from samples of PLWH. To this end, 500 ng of DNA from CD4 T cells of two different participants (P09 and P18, Supplementary Table S1) was first subjected to a pre-amplification step with six cycles, followed by UMI tagging as previously described. This was followed by various amplification conditions, tested in duplicate, varying both the number of PCR rounds and the number of cycles within each round (Supplementary Figure S3). The results of this experiment illustrate that only the condition with four consecutive rounds of 10 PCR cycles yielded enough template for downstream library preparation. Furthermore, this condition retained a high level of library complexity, illustrated by the patterns of bands on gel electrophoresis, which ranged from 700 bp to 10 kb (Supplementary Figure S3).

Next, we set out to determine whether high-accuracy HIV-1 genomes could be acquired by performing long-read sequencing of the J-Lat 8.4 PCR products and subsequent analysis using a custom version of the UMI pipeline developed by Karst *et al.* (25). Using an ONT R10.3 flow cell, reads with a median length of 9.5 kb were generated, in agreement with the expected amplicon length of J-Lat 8.4 DNA (Supplementary Figure S1C–H). The bioinformatics pipeline allowed for the construction of the UMI-tagged proviruses by binning the reads based on the observed terminal UMI pairs in the sequencing data (Figure 1A). During these steps, non-HIV reads were filtered out (removing non-specific amplicons) and aberrant UMI bins not meeting a fixed list of criteria (e.g. chimeras, read orientation bias) were excluded (Supplementary Figure S1B–H, for LIB01 (sequencing library containing the J-Lat 8.4 amplicon reads) ∼30% of all detected UMI pairs did not meet the criteria, however, they accounted only for 16% of all the binned reads). For each bin, a final proviral sequence was constructed by multiple rounds of polishing (racon, medaka) of the assigned raw reads. To assess the accuracy of the proviral UMI sequences, we compared each bin to a reference genome of the J-Lat 8.4 amplicon sequenced with Illumina. As more reads were assigned to a bin, an increase in the mean accuracy could be observed before reaching a plateau at 99.9% (Figure 1C). Bins with a coverage of at least 15 reads passed the Q30 (99.9% accuracy) threshold. At the Q30 threshold, an average of eight mismatches per 9.5 kb genome could be observed, while aberrant deletions and insertions were completely resolved (Figure 1D).

### Application of the assay on samples from PLWH and comparison to FLIPS

**Figure 2.**
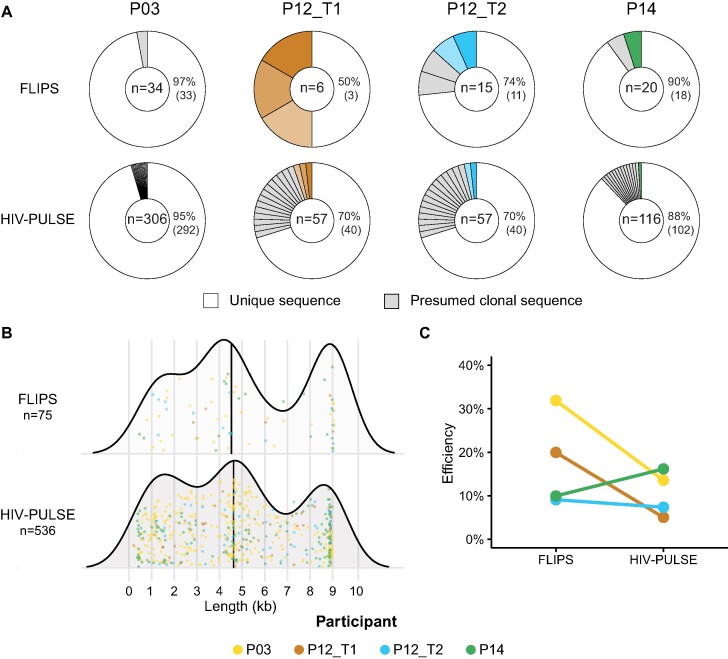
Benchmarking assays: novel HIV-PULSE vs gold standard FLIPS. **(A)** Donut plots displaying the fraction of unique and presumed clonal proviral sequences detected in each participant for both assays. The number of distinct proviruses generated by each assay is shown in the middle of each donut. The matching colored slices indicate the 6 out of 16 overlapping expansions of identical sequences (EIS) found to be clonal in both assays. A Fisher's exact test was used to compare the proportions of unique proviruses between both assays for each participant, none were significantly different (*P* = 1.00, *P* = 0.3716, *P* = 1.00, *P* = 1.00 for P03, P12_T1, P12_T2 and P14, respectively). **(B)** Size distributions of the observed proviral genome lengths for each assay. No significant difference was observed between both assays using a Mann–Whitney *U*-test (*P* = 0.08106). Each dot represents a single distinct provirus and is given a color for each participant. **(C)** For each assay and participant, the percentage of detected proviruses out of the total HIV-1 DNA reservoir size is shown. Assay efficiencies were compared for significance using a Mann–Whitney *U*-test (*P* = 0.3429).

To assess the performance of the HIV-PULSE assay on samples from ART-suppressed PLWH, and to benchmark it to a gold standard short-read sequencing assay, DNA from peripheral blood CD4 T cells from four individuals was subjected to both the HIV-PULSE and the FLIPS assay (Figure 2). This yielded a median of 18 FLIPS and 87 HIV-PULSE distinct proviruses per participant (Figure 2A, Supplementary Figure S4, Supplementary Tables S4 and S5). Of note, in the case of FLIPS, 10 out of 136 HIV-1 PCR amplicons failed to produce a correct HIV-1 genome during *de novo* assembly (Supplementary Figure S4A, median of 2.5 assembly failures per participant). Across both assays, a total of 16 overlapping expansions of identical sequences (EIS) were observed (Figure 2A). HIV-PULSE successfully identified 81% (13/16) of the overlapping EIS as being clonal, while FLIPS detected 37.5% of the EIS (6/16). However, this discrepancy is probably the result of the lower number of genomes assayed with FLIPS (∼5-fold). We compared the overlapping proviral sequences to estimate sequencing accuracy and link it to proviral classification. The mean accuracy was found to be 99.99% (Q40) for the megabinned proviruses, with residual errors observed in only four genomes due to homopolymeric regions (Supplementary Figure S4B, C). However, these errors did not affect the correct HIV-1 proviral classification.

Overall, comparing HIV-PULSE results to FLIPS data reveals (i) no significant differences between the proportions of sampled unique proviruses with each assay (Figure 2A; *P* = 1.00, *P* = 0.583, *P* = 1.00, *P* = 1.00 for P03, P12_T1, P12_T2 and P14, respectively) and (ii) no significant bias in the size distribution of the observed proviral genome lengths (Figure 2B, median lengths are 4620 for HIV-PULSE and 4531 for FLIPS, *P* = 0.0810). The efficiency of both methods was assessed by dividing the total number of detected proviruses (normalized to the assayed number of cells) to the total HIV-1 DNA per million CD4 cells as measured by ddPCR (Figure 2C). Slightly lower efficiencies were observed with the HIV-PULSE assay (mean of 11% opposed to 18% with FLIPS, *P* = 0.271), however, this measure is likely an underestimation as it does not account for clonality of the reservoir (true size of clones is missed).

To investigate the occurrence of potential recombination events during the pre-amplification or PCR amplification stages, a mixing experiment was performed with template from two participants (P03 and P12_T1). HIV-PULSE was performed in duplicate on a mix of 250 ng DNA of each participant extracted from CD4 T cells (500 ng total DNA per reaction). The results demonstrate the successful removal of chimeric reads by our bioinformatics pipeline (Supplementary Figure S5). No signs of chimera formation during pre-amplification were observed, as indicated by the clear separation between the proviral bins (*n* = 952 for replicate 1, *n* = 947 for replicate 2) from each participant in the phylogenetic trees in both replicates. Furthermore, the absence of any recombination detected between bins from P03 and P12_T1 by RDP4 (toolkit to detect recombination, see Material and Methods), further supports the lack of interindividual template recombinants (Supplementary Figure S5, Supplementary Table S6).

In conclusion, these results indicate that HIV-PULSE displays the required sensitivity for the amplification of NFL HIV-1 genomes from samples of ART-suppressed PLWH. Furthermore, both the accuracy and efficiency of the assay are on par with the FLIPS assay.

### HIV-PULSE enables high-throughput sequencing of NFL genomes in a large cohort of PLWH

**Figure 3.**
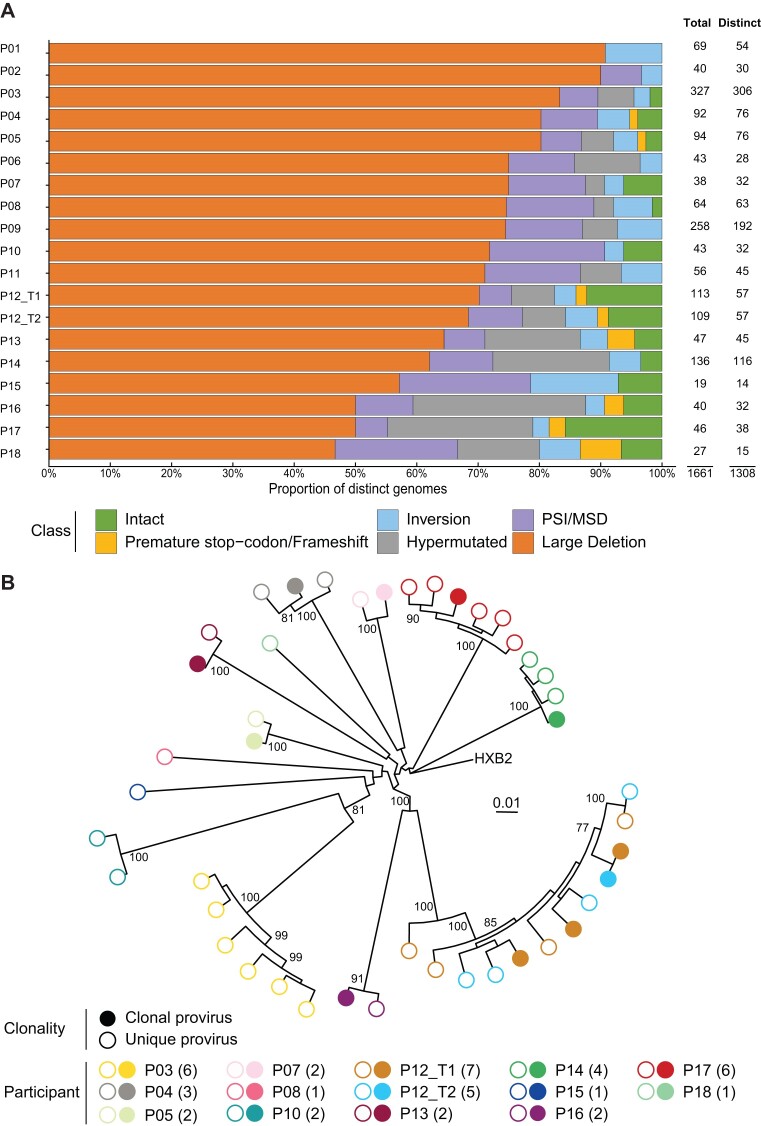
Proviral reservoir as assayed by the HIV-PULSE assay. **(A)** The proportions of different proviral classes observed among the distinct proviruses for each participant. On the right the number of total and distinct proviruses is displayed for each participant. **(B)** A phylogenetic tree including the distinct genome intact sequences. Each participant is shown as different colored dots, empty symbols indicate sequences only found once (unique, white insert) in a PCR replicate. The number of included intact genomes per participant are shown between brackets in the legend.

The assay was next applied to peripheral blood CD4 T cell DNA of 18 chronically treated PLWH (mean time on ART = 11.2 years, Supplementary Table S1). For each participant, the HIV-PULSE assay was performed with six PCR replicates of each 500 ng DNA input, yielding an average of 15 ± 3 distinct HIV-1 proviruses per replicate (range: 3–55). A mean PCR success rate of 97% was observed among the six PCR replicates based on agarose gel visualization (Supplementary Table S5). A total number of 1661 proviruses (1308 distinct) were retrieved across all participants (mean of 87 HIV-1 proviruses per sample, Supplementary Table S5, Supplementary Figure S6). Excluding the effect of clonal proliferation on infected cells, we looked at the presence of putatively intact genomes within the 1308 distinct proviruses. A mean proportion of 5% intact distinct genomes was found across the 19 samples, which corresponds to previously reported numbers (Figure 3A, Supplementary Table S4) (16–18). Putatively intact sequences found across multiple replicates, indicative of clonality, were seen in nine out of 14 participants with at least one distinct intact sequence (Figure 3B). As two collected samples belonged to the same individual (P12) taken 3 years apart, a longitudinal assessment of the reservoir composition could be performed. This revealed the persistence of 21 infected cell clones (two with an intact provirus) between the two sampled timepoints (Figure 3, Supplementary Figure S7). No significant differences were observed in both the yield (*P* = 0.662, 19 at T1 vs 18 at T2 mean distinct viruses per replicate, Figure 3A, Supplementary Table S2) and the observed mean pairwise distance among intact sequences between the two timepoints (*P* = 0.263, P12_T1: 0.0230 *n* = 7 vs P12_T2: 0.0197, *n* = 5).

Despite the inclusion of samples from individuals with a low total HIV-1 reservoir size (<500 total HIV DNA copies/million CD4, *n* = 3), we were still able to get an average of 55 total proviral sequences for those participants (range 27–92). A significant Spearman correlation between the HIV-PULSE yield and the reservoir size measured by total HIV DNA was observed (Supplementary Figure S8, *R* = 0.71, *P* = 1.5 × 10^15^). On average, the efficiency of HIV-PULSE per PCR replicate for these samples was 13% (95% CI [7.9, 17.6]), as calculated by dividing the number of detected distinct proviruses by the total number of input HIV-1 copies per PCR replicate (Supplementary Table S5). Nevertheless, this measure of efficiency is an underestimation, as it does not account for clones found within a replicate.

### HIV-PULSE detects proviruses of the translation-competent HIV-1 reservoir

**Figure 4.**
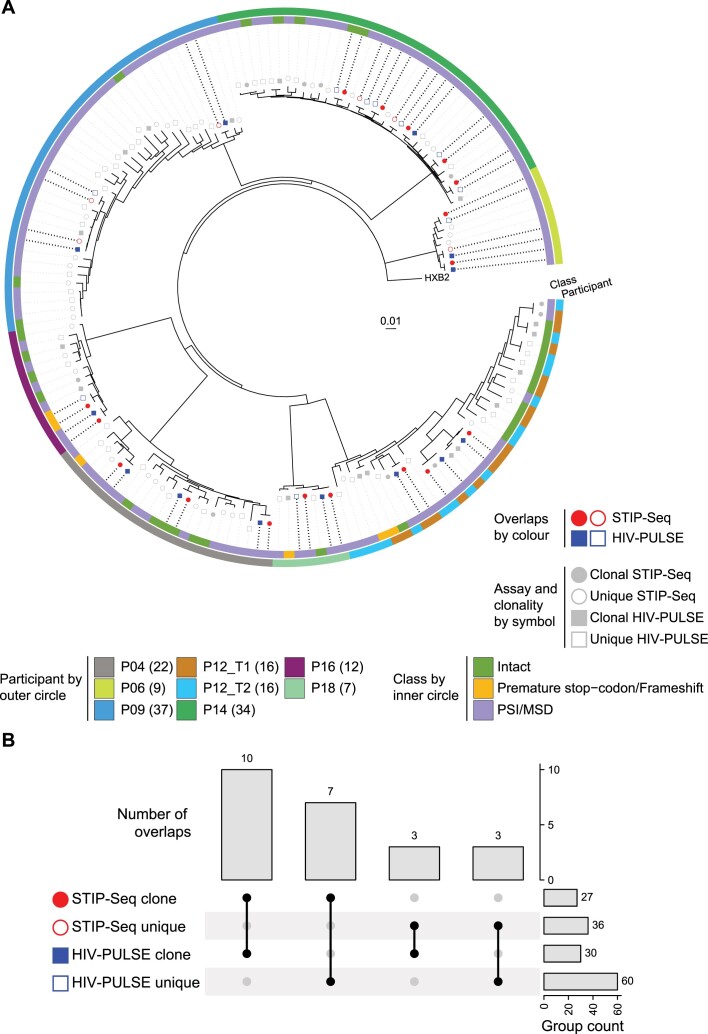
Benchmarking of HIV-PULSE vs STIP-Seq assay. **(A)** Phylogenetic tree including all distinct proviruses obtained with the HIV-PULSE (excluding sequences with inversions, large deletions and hypermutations) and STIP-Seq assays for eight participants. Symbols reflect the different assays, proviruses only recovered in a single assay are shown in grey while assay overlapping are shown in red (STIP-Seq) or blue (HIV-PULSE). Empty symbols indicate sequences were found once (unique, white insert) in that respective assay. The outer and inner circles indicate for each provirus, respectively, the participant origin and associated HIV-1 genome classification. The number of included genomes per participant are shown between brackets in the legend. **(B)** UpSet-plot visualizing the number of overlaps between clonal and unique proviruses recovered with each respective assays.

While the proviral reservoir consists of a heterogeneous mix of HIV-1 proviruses belonging to different classes, only a few can contribute to viral rebound and/or HIV-1 pathogenesis by inducing chronic immune activation (e.g. intact, PSI/MSD defect) (20,45,46). Of particular clinical interest, the translation-competent HIV-1 reservoir represents all proviruses that can produce viral proteins following maximal stimulation, consequently enriching for replication-competent proviruses (45). In this regard, we next set out to evaluate whether HIV-PULSE can capture proviruses that belong to the translation-competent reservoir, by comparing HIV-PULSE data (*n* = 8 participants) to STIP-Seq data (Figure 4A). On average per individual, 69% (95% CI [45, 92]) of the translation-competent STIP-Seq clones were detected with HIV-PULSE (as unique or clone). This corresponds to a total of 17 overlaps among both assays, of which 10/17 (59%) were also identified as a clone by HIV-PULSE, as they were detected across multiple replicates from the same participant (Figure 4B). Additionally, 17 clones were found in the HIV-PULSE data but not sampled with STIP-Seq (6/17 intact), which could be due to the integration of those proviruses at a chromosomal location in regions with features (e.g. heterochromatin) associated with HIV-1 transcriptional latency or a location less prone to be induced by latency reversing agents or by the more limited number of proviruses assessed with STIP-Seq compared to HIV-PULSE (47,48). No disagreements in proviral classification were observed in all 23 assay overlapping proviruses, with an average reported sequence accuracy of 99.99% (Supplementary Figure S9). In conclusion, HIV-PULSE reliably picks up proviruses that belong to the translation-competent reservoir, which is of high importance for applicability in a clinical setting.

## Discussion

To achieve an HIV-1 cure, a comprehensive understanding of the persisting viral reservoir is crucial. Over recent years, the application of HIV-1 NFL sequencing assays has increased our knowledge of certain key aspects, such as the proviral genomic composition and reservoir dynamics within PLWH. Still, all these results have been obtained through labor-intensive assays relying on limiting dilution and subsequent one-by-one sequencing of proviral genomes, limiting their application in large-scale studies. Here, we present the HIV-PULSE assay, allowing for a scalable, high-throughput assessment of the proviral HIV-1 reservoir. The use of dual barcodes removes the need for limiting dilution, allowing the amplification of multiple proviral HIV-1 templates during long-range NFL PCR on bulk DNA, while overcoming the inherently low single-read accuracy of long-read sequencing technologies. Benchmarking against the gold standard FLIPS method revealed comparable accuracy and efficiency, though notable differences in terms of throughput and associated costs are apparent. An overview of the approximated cost (in USD) per proviral sequence for each method indicates a 10-fold price reduction in favor of HIV-PULSE (Supplementary Table S7). Aside from a clear cost benefit, eliminating the limiting dilution step and sampling multiple proviruses out of a single PCR reaction offers a more high-throughput approach to NFL HIV-1 reservoir characterization. To illustrate, at least 52 96-well FLIPS PCR plates at limiting dilution would be required to obtain the equivalent of 1661 total HIV-PULSE proviruses (Supplementary Table S7). Furthermore, inherent to the use of short-read NGS, FLIPS requires a *de novo* assembly step, which sometimes fails when resolving more complex genomes.

In this study, we applied HIV-PULSE to peripheral blood samples from a cohort of 18 PLWH on chronic ART to study the composition of their HIV-1 viral reservoir. Out of the 1308 distinct proviruses detected, ∼5% were deemed genome-intact, agreeing with earlier reports on PLWH on chronic ART (16). We compared the HIV-PULSE assay to the STIP-Seq assay for eight participants, showing that HIV-PULSE efficiently picked up translation-competent proviruses, detecting 69% of the clonal sequences found with STIP-Seq. Interestingly, HIV-PULSE detected additional putatively intact proviral genomes that were not detected with the STIP-Seq assay, potentially representing hard-to-reactivate proviruses in a deep state of latency. While the HIV-PULSE assay does not enable a specific enrichment of the translation-competent reservoir, these findings make a case for HIV-PULSE as a tool to perform qualitative in-depth characterization of the functional reservoir dynamics in response to curative interventions during clinical trials. The high-throughput and cost-efficient nature of the HIV-PULSE assay makes it an attractive method for use within large-scale clinical studies of the HIV-1 reservoir with applications ranging from performing a longitudinal phylogenetic analysis of the proviral reservoir, screening multiple samples from the same individual for compartmentalization across different tissues, drug-resistance screening and bNAb epitope mapping. Indeed, implementing a qualitative NFL approach in clinical trial settings could help to check whether participants are eligible by excluding pre-existing resistance to a compound of interest or to assess immune escape following the intervention (49–51).

The adoption of long-read sequencing technologies for amplicon sequencing has historically been taken aback by the poor single-read accuracy. Notwithstanding these initial reservations, others have been developing long-read assays to characterize different aspects of the HIV-1 reservoir over the last couple of years. Pooled CRISPR Inverse PCR sequencing (PCIP-seq) allows to study both the integration site and the associated provirus using a targeted enrichment and inverse PCR strategy (52). While the collected data is certainly informative, the approach is hampered by limited sensitivity (3.2%), inadequate proviral coverage for accurate genome assembly and its reliance on the design of a custom pool of CRISPR guide RNAs for each participant. In comparison, HIV-PULSE has an improved sensitivity (13%), produces high-accuracy NFL genomes, and does not rely on individualized primer designs, although information on the genomic location of the integrated provirus is missing. Another group developed NanoHIV, a bioinformatics tool to construct HIV-1 consensus sequences from long-read ONT data (53). This follows a reference mapping-based strategy with consecutive mappings to refine the original draft and deal with variable genomic regions. To compare the performance, they generated consensus genomes of NFL amplicons (acquired via nested NFL PCR performed at limiting dilution) and performed sequencing with both ONT and NGS Illumina. The authors report a mean accuracy of 99.4% (or 54 errors in a 9 kb genome), considerably lower than the megabin accuracy of 99.99% (or one error in a 9 kb genome) reported with our bioinformatics pipeline. Two studies describe protocols to amplify and sequence different genomic regions with accuracies up to 99.9% from virions in plasma samples from viremic individuals. While one relies on circular consensus sequencing reads with PacBio technology to obtain 2.6 kb full-length *env* sequences (54), the other Multi-read Hairpin Mediated Error-Correction Reaction (MrHAMER) method targets a 4.6 kb *gag-pol* region followed by sequencing on a MinION ONT device (55). Despite showing great promise, the aforementioned strategies have not been applied to the more challenging setting of HIV-1 reservoir, which requires several orders of magnitude greater sensitivity.

We do acknowledge some limitations to the HIV-PULSE assay. First, we recognize that enriching HIV proviral templates by means of PCR introduces certain biases associated with NFL HIV PCR-based assays. The reliance on specific primer designs could influence assay performance as sequence polymorphisms within the viral genome might potentially lead to dropouts or lower amplification efficiency (56). The former can partially be mitigated by using alternative primers tailored to viral subtypes or even individual specific designs (17–19). Furthermore, White *et al.* have reported biases in long-range amplification of short versus full-length proviral templates, which limit the accuracy of quantitative viral reservoir measurements based on NFL sequencing (56). Second, the inclusion of the pre-amplification step to ensure efficient tagging and enrichment impedes the accurate quantification of reservoir clonality, as the dual UMI tags are only incorporated after the initial pre-amplification cycles. However, by performing multiple PCR replicates, we were still able to identify most clonal populations throughout this study. Further research into increasing the efficiency of the UMI tagging step would be needed to omit the pre-amplification step. Despite the limitations of HIV-PULSE in terms of accurate reservoir quantification, the assay can be valuable in areas where quantification does not matter, such as the aforementioned HIV-1 phylogenetics, drug resistance screening, and bNAb epitope mapping. Third, although we cannot completely rule out the possibility of chimera formation during the initial pre-amplification step, we consider it highly unlikely. PCR-induced chimeric products are typically observed in reactions with a high number of PCR cycles, while the pre-amplification stage in HIV-PULSE only uses six or fewer cycles, minimizing the likelihood of recombination (57). Furthermore, as supported by our experiments, the inclusion of dual UMIs allows for the detection and exclusion of chimeric reads introduced during later PCR stages, increasing the reliability of the HIV-PULSE data. Fourth, we successfully applied the HIV-PULSE assay on our cohort, yet samples from PLWH in different settings might be more challenging. As some steps of the analysis workflow rely on the sequence diversity to cluster identical bins into megabins to deconvolute the effect of pre-amplification, proviral reservoirs with lower intra-host sequence diversity (e.g. early ART initiation) could limit the success of this approach.

In conclusion, the HIV-PULSE assay presents itself as a promising HIV-1 NFL proviral sequencing method that enables scalable, high-throughput characterization of the proviral reservoir, while retaining sequencing accuracy comparable to HIV-1 NFL assays currently used in the field. We are convinced that the HIV-PULSE assay will be a valuable asset in advancing our understanding of the composition and dynamics of the viral reservoir during future basic and translational HIV-1 research.

## Supplementary Material

gkad790_Supplemental_Files

## Data Availability

The HIV-1 proviral sequences within this article are available in GenBank at https://www.ncbi.nlm.nih.gov/genbank/, and can be accessed under MW881651–MW881678 and OQ596824–OQ596881 and OR245577–OR246884. The sequencing data within this article are available in the Sequence Read Archive (SRA) at https://www.ncbi.nlm.nih.gov/sra/ under PRJNA925880. The bioinformatics pipeline within this article is available at https://doi.org/10.5281/zenodo.8102644.

## Notes

Present address: Noah Bonine, Laboratory for Translational Oncogenomics and Bioinformatics, VIB-UGent Center for Medical Biotechnology, Department of Biomolecular Medicine, Ghent University, 9000 Ghent, Belgium.

Present address: Basiel Cole, Translational Cell and Tissue Research Unit, Department of Imaging and Pathology, KU Leuven, 3000 Leuven, Belgium.
