# Supplementary material for: HIV-PULSE: a long-read sequencing assay for high-throughput near full-length HIV-1 proviral genome characterization
Source: Nucleic Acids Res. 2023 Oct 11;51(20):e102. doi: 10.1093/nar/gkad790 (PMC10639044; doi:10.1093/nar/gkad790)
Supplement: gkad790_Supplemental_Files [file gkad790_supplemental_files.zip › Supplemental_data_20230703_NAR_review.pdf]

Supplemental Table 1. Clinical characteristics of participants.

Participant ID	Age (years)	Gender	CD4/CD8 ratio	NADIR	Subtype	VL	Time since infection (years)	Time to ART (years)	ART duration (years)	FLIPS	STIP-Seq	HIV-PULSE
P01	48	M	0.5	361	B	<20	13.6	2.6	11.1			x
P02	32	M	0.8	405	B	<20	8.7	2.9	5.8			x
P03	48	M	0.6	118	B	<20	11.5	3.0	8.4	x		x
P04	50	M	0.5	488	B	<20	16.4	15.2	1.2		x	x
P05	58	M	0.8	182	B	<20	17.0	0.7	16.3			x
P06	55	M	0.6	327	B	<20	21.3	3.0	18.3		x	x
P07	49	M	0.6	294	B	<20	19.8	3.4	16.4			x
P08	40	M	1.3	350	B	<20	8.4	1.1	7.3			x
P09	60	M	0.8	179	B	<20	30.1	5.8	24.3		x	x
P10	32	M	0.8	395	B	<20	NA	NA	1.4			x
P11	54	M	0.9	231	B	<20	15.6	2.8	12.8			x
P12_T1	52	M	0.3	44	B	<20	NA	9.8	15.5	x	x	x
P12_T2	55	M	0.3	44	B	<20	NA	9.8	18.5	x	x	x
P13	37	M	1.1	382	B	<20	7.5	2.4	5.1			x
P14	50	M	0.9	171	B	<20	NA	NA	18.2	x	x	x
P15	62	M	0.8	196	B	<20	11.6	3.0	8.7			x
P16	38	M	0.9	492	B	<20	8.2	6.5	1.7		x	x
P17	31	M	1.0	NA	B	<20	11.7	4.2	7.5			x
P18	56	M	0.6	98	B	<20	NA	NA	14.7		x	x
Median values	*50*		*0.8*	*263*			*12.7*	*3.0*	*11.1*			

Supplemental Table 2. List of primers used throughout the study.

See Excel file.

Supplemental Table 3. Estimates for minimal required read depth for ONT sequencing experiments.

	HIV-PULSE minimum read depth requirements			
**Total HIV DNA/million CD4**	*350*	*1000*	*4500*	**Means**
Bins passing QC for 6 replicates	1,000	1,200	5,400	*2,533*
*Average reads required for bins passing QC **	60,000	72,000	324,000	*152,000*
Total reads per participant †	150,000	180,000	810,000	*380,000*

** The number of reads needed to meet the Q30 target for all bins passing QC is calculated as 60 times the number of bins. To ensure accurate Q30 coverage depth, a minimum of 15 reads per bin is required. However, to account for coverage bias caused by variations in bin sequence lengths, an average of 60 reads per bin is used. This ensures that longer fragments, which may have lower coverage, still meet the minimum requirement of 15 reads per bin. (Supplemental Figure 6 demonstrates a 4-fold lower coverage between bins with a median length of 4000 bp compared to bins containing longer sequences of 9000 bp.)*

† The total reads are determined by correcting the required average reads for background, non-binned and chimeric proportions (= average reads / 0.4).

Supplemental Table 4. Counts of distinct proviruses per proviral category for each assay and participant.
See Excel file.

Supplemental Table 5. Performance results of the HIV-PULSE assay.

Participant ID	total HIV DNA/million CD4	Pre-amplification cycles	PCR replicates	PCR replicates sequenced	PCR succes rate[1]	Number of total proviruses	Number of distinct proviruses	Mean distinct proviruses per replicate[2]	Mean standard devation of distinct proviruses per replicate	Mean distinct proviruses/HIV copies for 500 ng DNA input[3]
P01	653	6	6	6	100%	69	54	12	2	17%
P02	NA	6	6	6	100%	40	30	7	3	NA
P03	4869	5	6	6	100%	327	306	55	6	9%
P04	373	6	6	6	100%	92	76	15	2	18%
P05	1589	6	6	6	100%	94	76	16	4	12%
P06	NA	6	6	6	100%	43	28	7	2	NA
P07	983	6	6	5	83%	38	32	8	4	8%
P08	1085	6	6	6	100%	64	63	11	2	9%
P09	4392	5	6	5	83%	258	192	52	6	15%
P10	920	6	6	5	83%	43	32	9	2	11%
P11	1441	6	6	6	100%	56	45	9	4	9%
P12_T1	4537	5	6	6	100%	113	57	19	3	3%
P12_T2	2988	5	6	6	100%	109	57	18	1	2%
P13	322	6	6	6	100%	47	45	8	2	9%
P14	1699	6	6	6	100%	136	116	23	1	41%
P15	764	6	6	6	100%	19	14	3	2	6%
P16	NA	6	6	6	100%	40	32	7	4	NA
P17	1182	6	6	6	100%	46	38	8	2	20%
P18	366	6	6	6	100%	27	15	5	1	15%
Median values	*1134*			*6*	*100%*	*56*	*45.0*	*9.3*	*2.2*	10%

[1] *The fraction of sequenced PCR replicates out of performed PCR replicates.*

[2] *The average of detected distinct proviral bins observed over all replicates.*

[3] *Efficiency of HIV-PULSE per PCR replicate was calculated by dividing the mean number of detected distinct proviruses by the number of total HIV-1 DNA present in 500 ng DNA input.*

Supplemental Table 6. Analysis results from the HIV-PULSE recombination experiment.

	Replicate 1	Replicate 2
Total bins passing QC	952	947
Bins belonging to P03	795	711
Bins belonging to P12_T1	157	236
Potential recombination events detected by RDP4 (at least 5 out of 7 methods)	17	13
True recombination events (after manual inspection)	0	0

Supplemental Table 7. Estimated costs per sequenced virus for FLIPS and HIV-PULSE.

	FLIPS			HIV-PULSE		
	Cost per sequence (USD)	Cost for 1661 sequences by 52x96 rx (USD)	Time (h)	Cost per sequence (USD)	Cost for 1661 sequences by 111 rx (USD)	Time (h)
PCR amplification	11	18405	15	1.0	1662	12
Library Preparation	21	35409	3	0.7	1092	3
Sequencing	19	32282	24	2.4	4039	24
**Total**	52	86096	42	4	6793	39

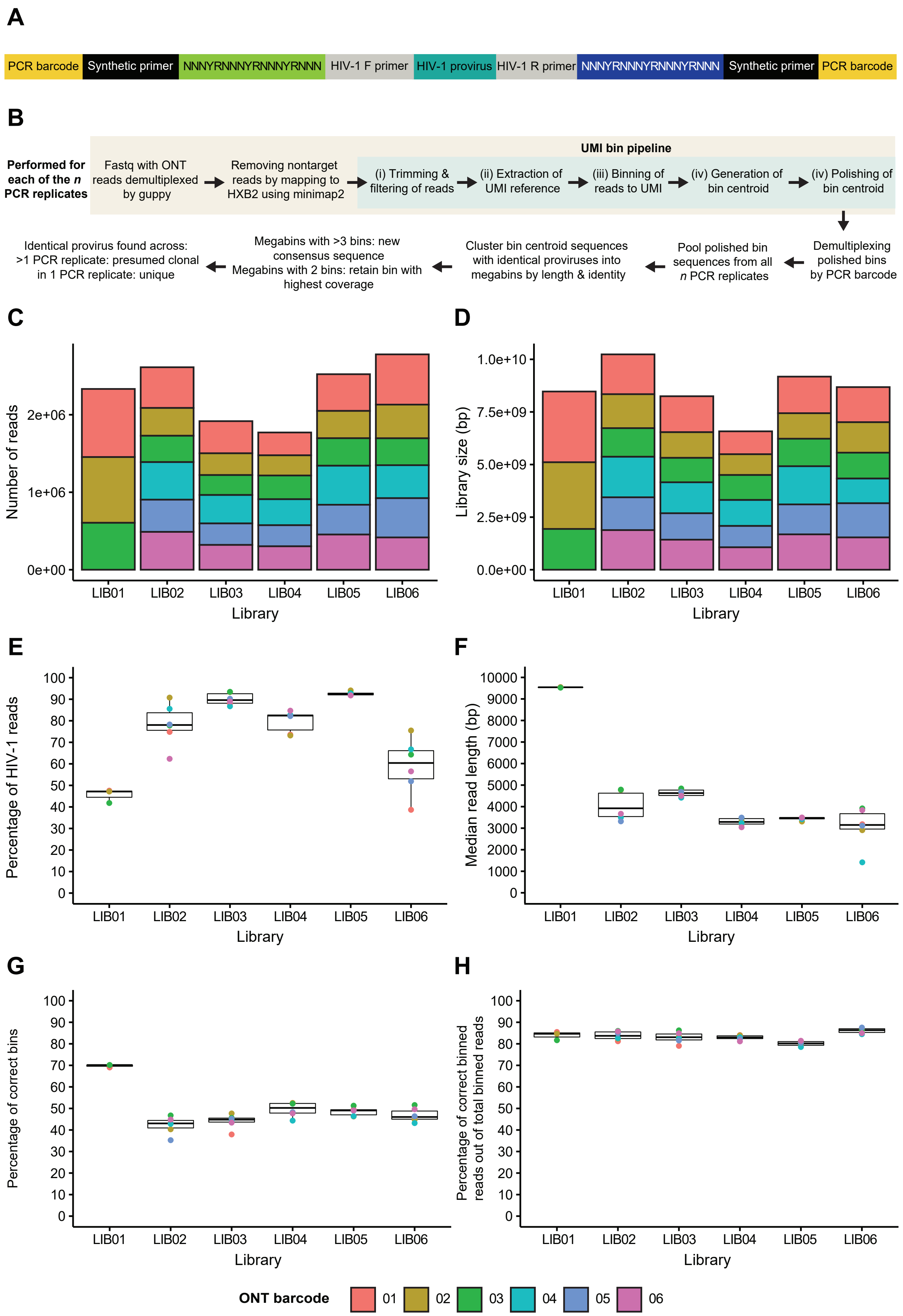

Supplemental Figure 1. HIV-PULSE assay details and performance. (A) Visual presentation of the HIV-PULSE read construct layout. (B) Schematic representation of the bioinformatics workflow to analyze HIV-PULSE data. (C) Number of total reads for each HIV-PULSE sequencing run (LIB1 contained J-Lat 8.4 amplicon data, from LIB2 onwards clinical samples). (D) Sequencing library size (in base pairs) for each HIV-PULSE sequencing run. (E) Percentage of HIV-1 reads out of the total reads for each HIV-PULSE sequencing run. (F) Median read length of HIV-1 reads for each HIV-PULSE sequencing run. (G) Percentage of bins deemed correct out of the total detected bins for each HIV-PULSE sequencing run. (H) Percentage of reads belonging to correct bins out of the total number of binned reads for each HIV-PULSE sequencing run.

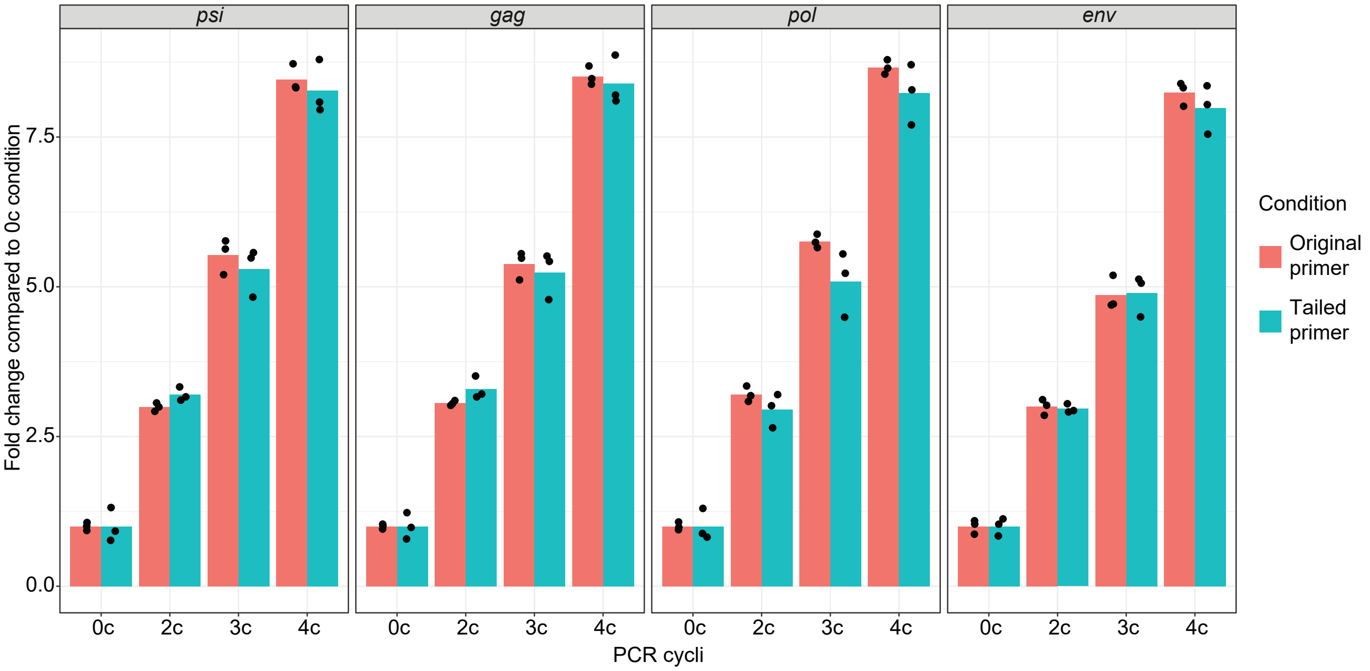

Supplemental Figure 2. Fold changes of HIV copy numbers after tagging PCR using primers without and with additional sequences to their tails. The tagging PCR was performed using 250 ng of a 10% diluition of J-Lat 8.4 DNA in Jurkat DNA with a varying number of cycles. Each condition (without and with tail) was performed with three replicates. Amplified products were measured using a multiplex dPCR targetting different HIV-1 genomic regions. Fold changes were calculated by comparing the measured HIV-1 copy numbers to the non-cycled (0c) condition.

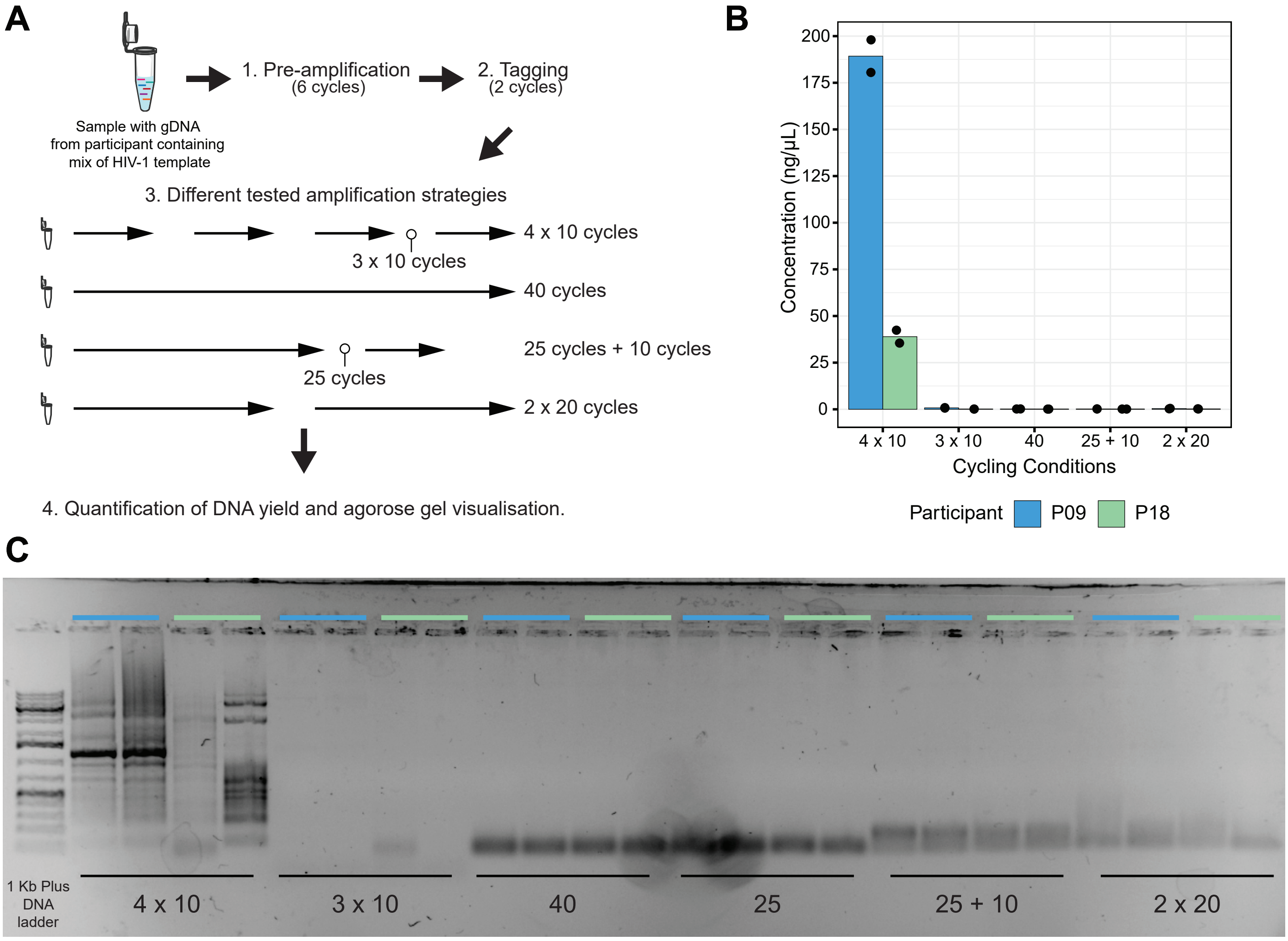

Supplemental Figure 3. HIV-PULSE amplification strategy optimisation. (A) Visual presentation of the experimental setup to test different amplification conditions. Genomic DNA from a participant went through the regular pre-amplification (6 cycles) and tagging (2 cycles) stages. Next, four different conditions were tested varying both the number of PCR rounds and the number of cycles within each round. Final PCR products were analyzed for yield by DNA quantifcation and agarose gel visualisation. In addition, two intermediate steps (3 x 10 cycles and 25 cycles) were also sampled. Each condition was performed in duplicate with experiments repeated on DNA from two different participants. The illustration of the microtube was obtained from SMART (Servier Medical Art; http://smart.servier.com/), licensed under a Creative Common Attribution 3.0 Unported license (https://creativecommons.org/licenses/by/3.0/). (B) Measured DNA concentrations for each condition per participant. No results for the 25 cycles condition are shown as no material for quantification remained. (C) Agarose gel results for all tested PCR conditions. Colored bars mark lanes belonging to each participant.

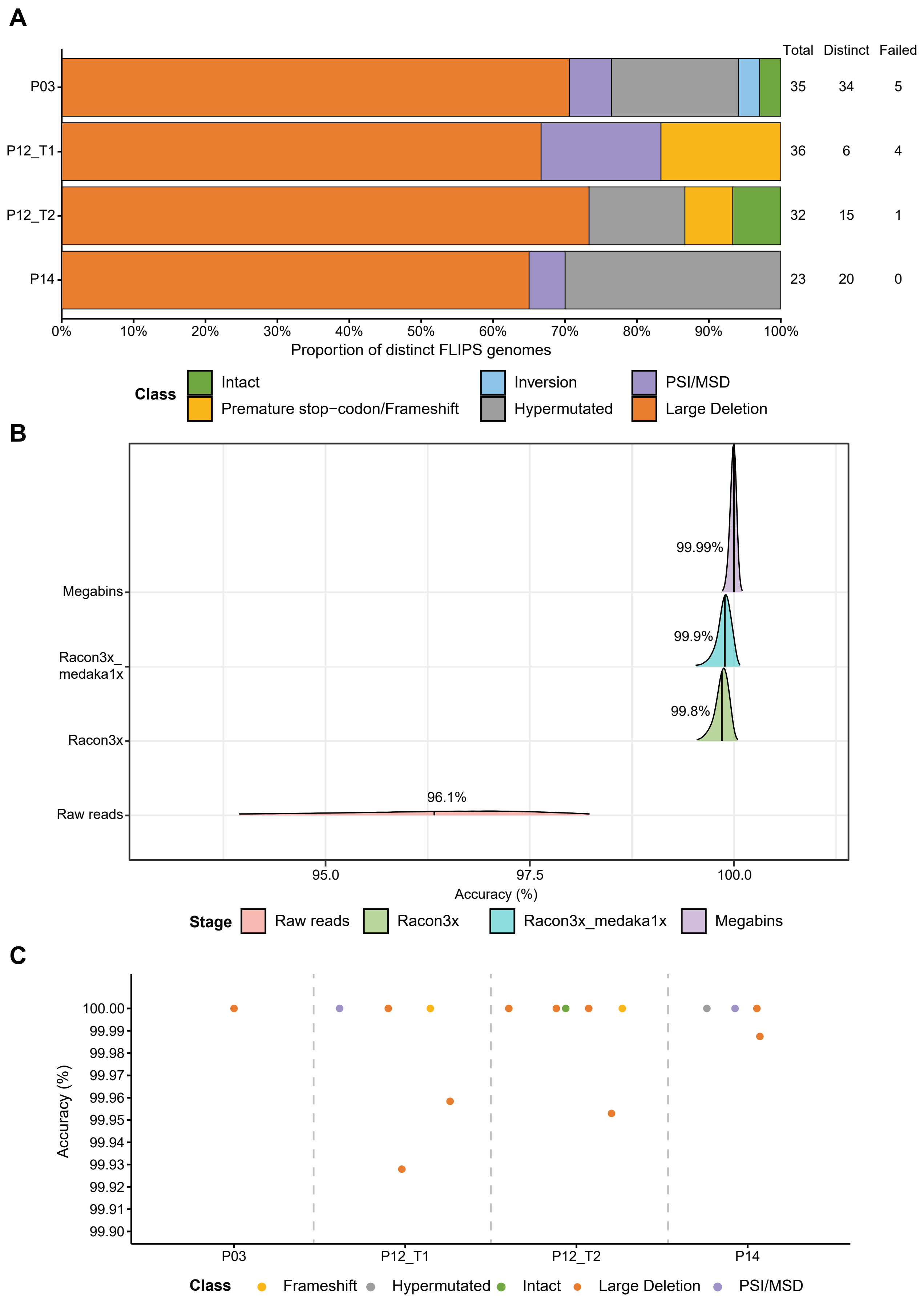

Supplemental Figure 4. Proviral reservoir as assayed by FLIPS. (A) The proportions of different proviral classes observed among the distinct FLIPS proviruses for each participant. On the right the number of total and distinct proviruses, including proviruses that failed during de novo assembly is displayed for each participant (B) Distribution of the accuracy rates for all overlapping proviruses at different stages of the bioinformatics pipeline. The raw reads indicate the single-read accuracy (n= 232,131), racon3x and racon3x_medaka1x depict the HIV-PULSE bins (n=2,668) and megabins consists of the clustered HIV-PULSE bins (n=16). (C) Accuracy rates of overlapping proviruses detected with HIV-PULSE assay compared to their FLIPS Illumina reference counterpart. The color indicates the proviral genome classification by the HIV-PULSE assay for each respective provirus.

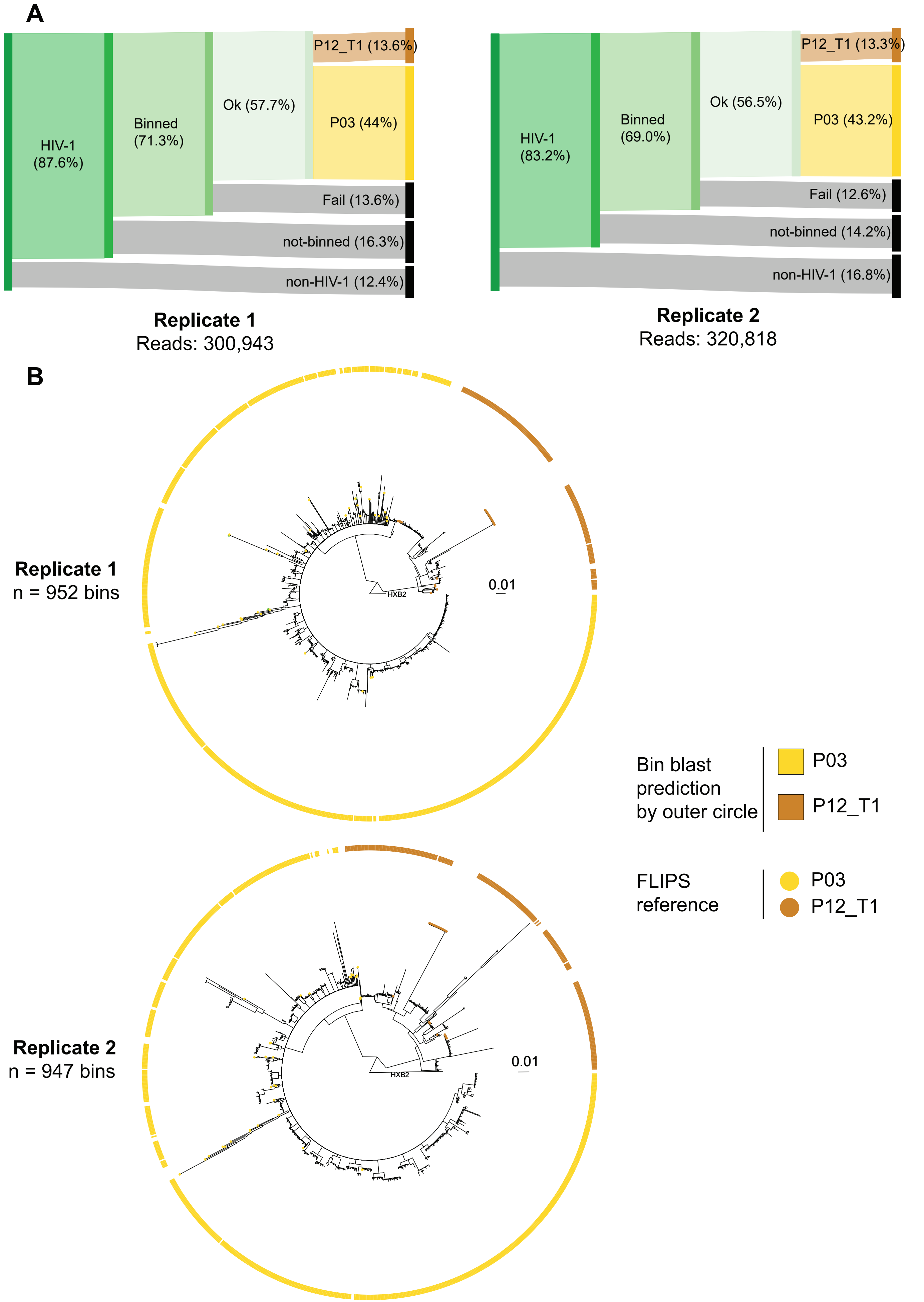

Supplemental Figure 5. Recombination analysis of HIV-PULSE data using mixed input template. (A) Read distributions showing their relative proportion during each stage of the bioinformatics workflow (respectively the reads mapping to HIV-1, part of a detected bin, passing bin QC filtering, assigned to participant part of the mixed template). (B) Phylogenetic trees of proviral HIV-PULSE bins passing the QC filtering and FLIPS reference genomes (marked by circles) from both participants (excluding inversions). Outer circle displays assigned participant identified by blast for each bin.

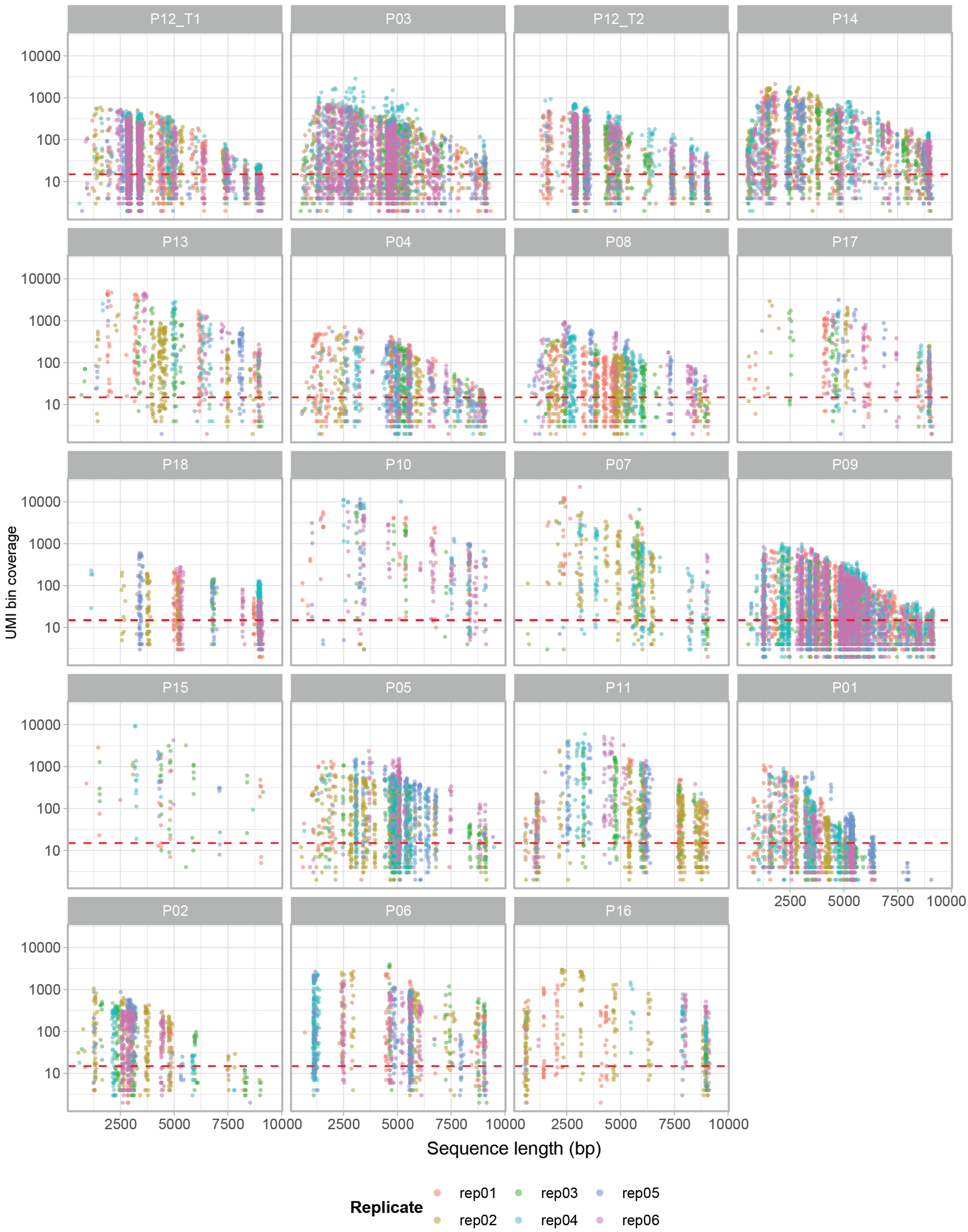

Supplemental Figure 6. Bin coverage in function of amplicon length for all individuals. Each dot represents a single HIV-PULSE bin and is given a color based on the PCR-replicate. The dashed red line indicates the Q30 (99.9%) bin accuracy threshold.

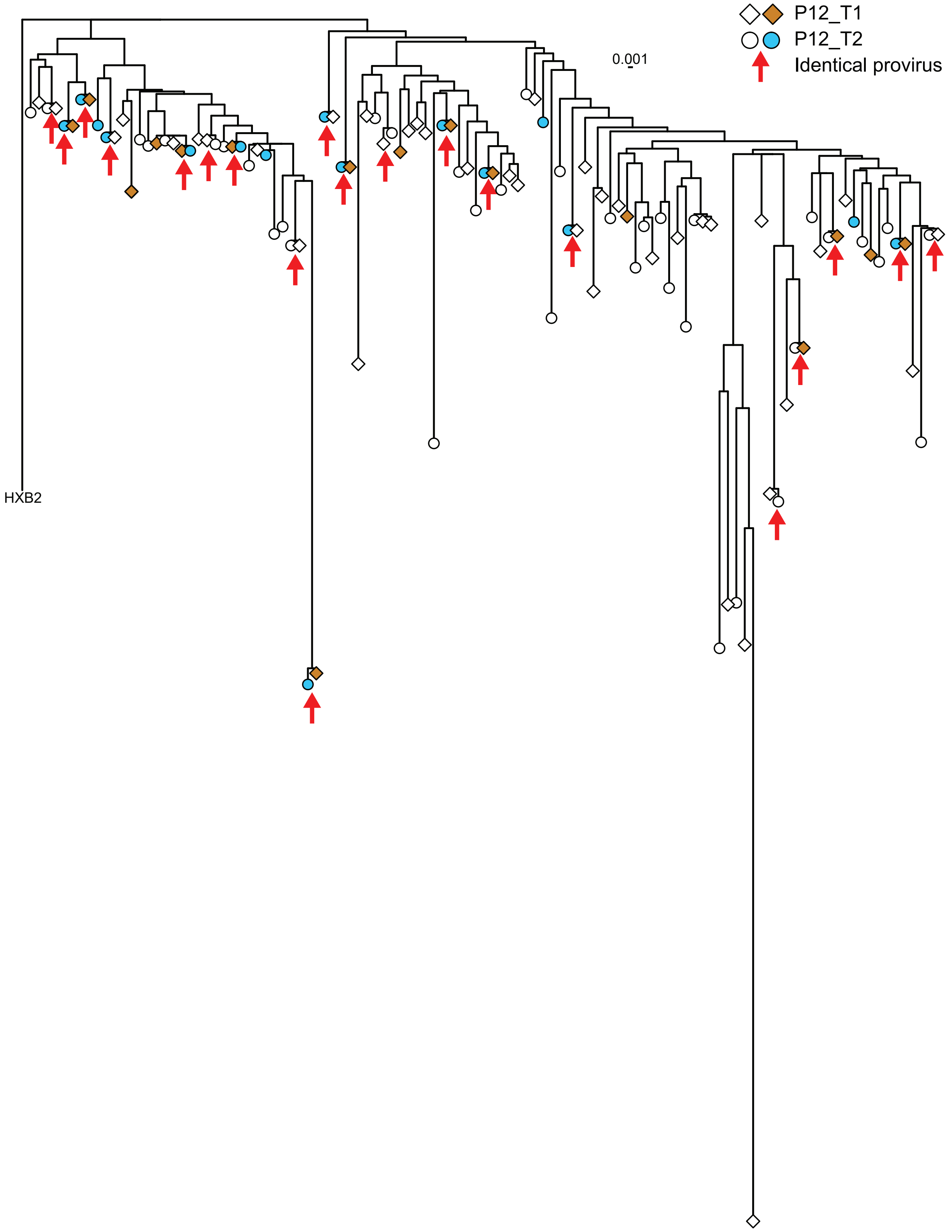

Supplemental Figure 7. Comparison of longitudinal sequencing data for P12. Phylogenetic tree of HIV-PULSE proviruses from P12 sampled at different timepoints (3 year interval). Tree was rooted against HXB2 and inversions were excluded (excluding 1 of the 21 timepoint overlapping clonal sequences). The symbols indicate the first (diamond) and second (circle) sampling timepoint while colors indicate whether the provirus was detected as clonal by the HIV-PULSE assay at that timepoint. The red arrows highlight identical proviral sequences detected at both timepoints.

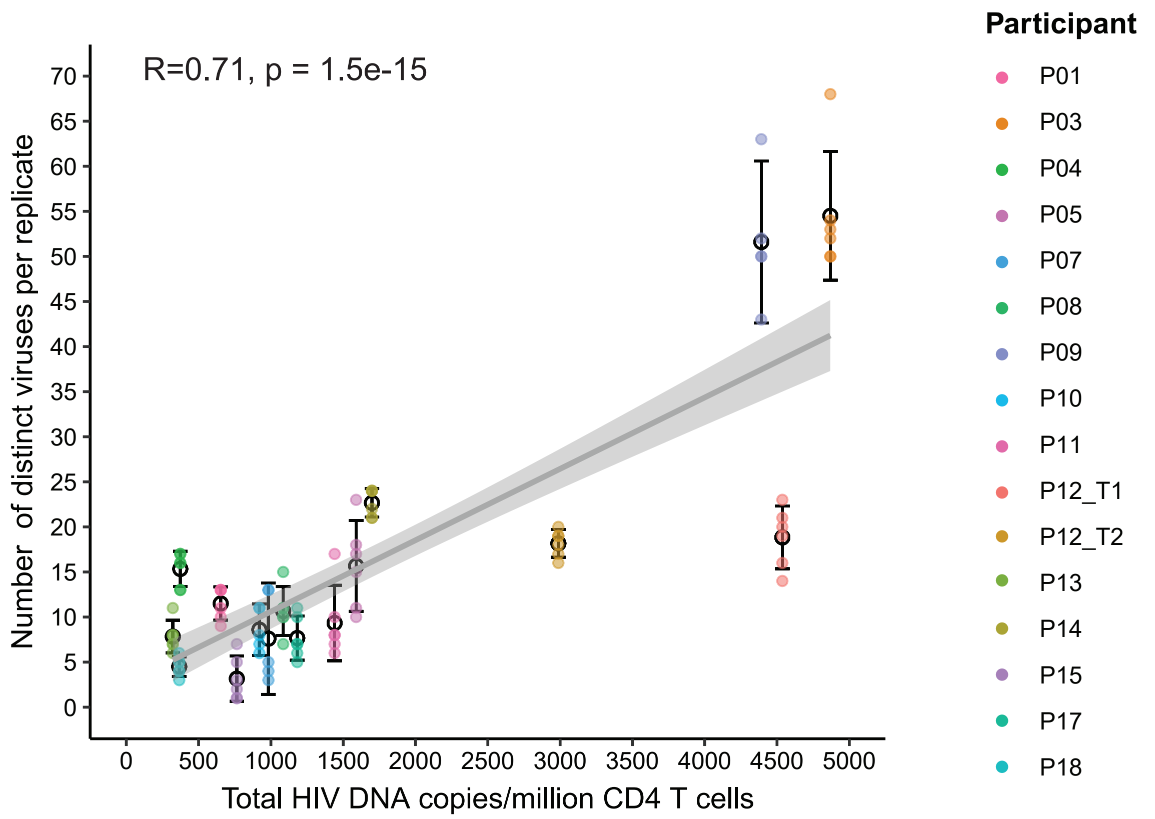

Supplemental Figure 8. Correlation between the number of distinct HIV-1 proviruses per PCR replicate and the total HIV-1 DNA reservoir size. For each participant, the number of distinct viruses for each sequenced PCR replicate are shown with the averages indicated as empty circles. A Spearman correlation (R=0.71, *p*=1.5*1015) is made between the mean number of distinct and total HIV-1 DNA copies/million CD4 cells as measured by ddPCR.

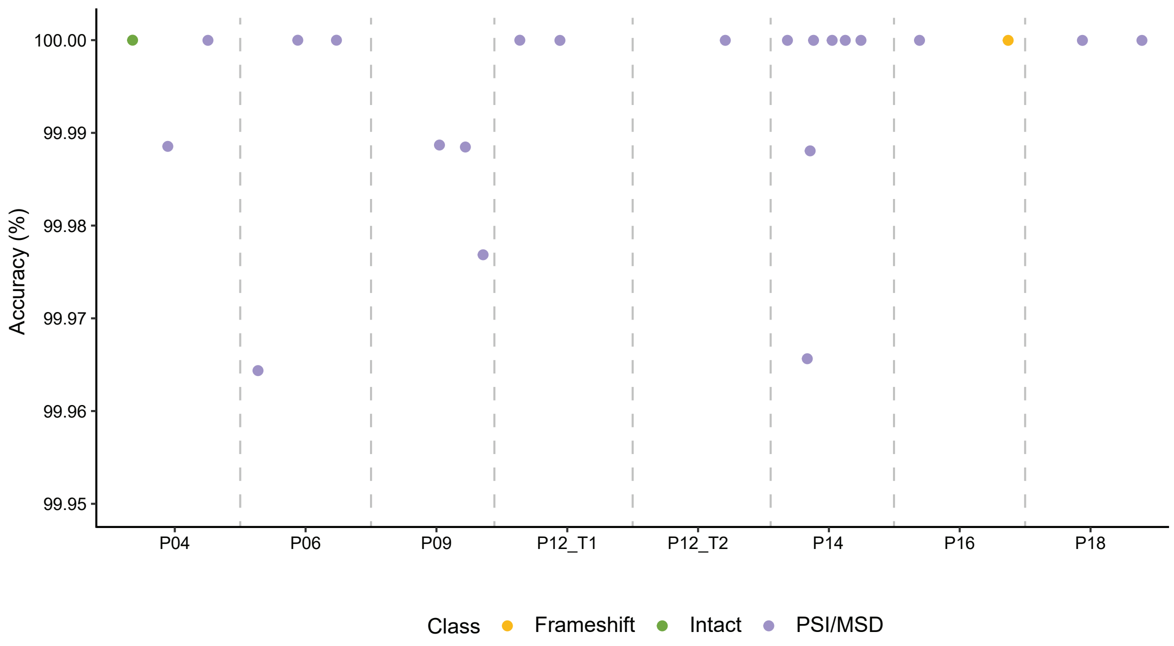

Supplemental Figure 9. HIV-PULSE genome accuracy compared to STIP-Seq reference sequences. Accuracy rates of overlapping proviruses detected with HIV-PULSE assay compared to their STIP-SEQ Illumina reference counterpart (n=23). The color indicates the proviral genome classification by the HIV-PULSE assay for each respective provirus.
